# Interaction of glycosphingolipids GD3 and GD2 with growth factor receptors maintains breast cancer stem cell phenotype

**DOI:** 10.18632/oncotarget.17665

**Published:** 2017-05-07

**Authors:** Yuh-Jin Liang, Chen-Yu Wang, I-An Wang, Yi-Wen Chen, Li-Tzu Li, Chuang-Yu Lin, Ming-Yi Ho, Tsung-Lung Chou, Ya-Hui Wang, Shih-Pin Chiou, Yu-Ju Lin, John Yu

**Affiliations:** ^1^ Institute of Stem Cell and Translational Cancer Research, Chang Gung Memorial Hospital at Linkou, Taoyuan, Taiwan; ^2^ Department of Physiology and Pharmacology, College of Medicine, Chang Gung University, Taoyuan, Taiwan; ^3^ Institute of Cellular and Organismic Biology, Academia Sinica, Taipei, Taiwan

**Keywords:** glycosphingolipid, ganglioside, breast cancer stem cell, GD3 synthase, glycosyltransferase

## Abstract

Many studies have suggested that disialogangliosides, GD2 and GD3, are involved in the development of various tumor types. However, the functional relationships between ganglioside expression and cancer development or aggressiveness are not fully described. GD3 is upregulated in approximately half of all invasive ductal breast carcinoma cases, and enhanced expression of GD3 synthase (GD3S, alpha-N-acetylneuraminide alpha-2,8-sialyltransferase) in estrogen receptor-negative breast tumors, was shown to correlate with reduced overall patient survival. We previously found that GD2 and GD3, together with their common upstream glycosyltransferases, GD3S and GD2/GM2 synthase, maintain a stem cell phenotype in breast cancer stem cells (CSCs). In the current study, we demonstrate that GD3S alone can sustain CSC properties and also promote malignant cancer properties. Using MALDI-MS and flow cytometry, we found that breast cancer cell lines, of various subtypes with or without ectopic GD3S-expression, exhibited distinct GD2/GD3 expression profiles. Furthermore, we found that GD3 was associated with EGFR and activated EGFR signaling in both breast CSCs and breast cancer cell lines. In addition, GD3S knockdown enhanced cytotoxicity of the EGFR-inhibitor gefitinib in resistant MDA-MB468 cells, both *in vitro* and *in vivo*. Based on this evidence, we propose that GD3S contributes to gefitinib-resistance in EGFR-positive breast cancer cells and may be an effective therapeutic target in drug-resistant breast cancers.

## INTRODUCTION

Glycosphingolipids (GSLs) are amphiphilic membrane lipids consisting of a polar oligosaccharide chain attached to a hydrophobic sphingosine-containing ceramide lipid moiety. They are expressed ubiquitously in animal cell membranes where they mediate cell adhesion and signal transduction via lipid rafts [2]. Clusters of GSLs on the cell surface membrane interact with functional membrane proteins such as growth factor receptors (GFRs), integrins, and non-receptor cytoplasmic protein kinases to form glycosynaptic domains, which are involved in regulation of cell adhesion, growth, and motility [3, 4].

Gangliosides are sialic acid-containing GSLs. The disialogangliosides GD3 and GD2 have been characterized as oncofetal markers [5]. In mice and other mammals, they are expressed primarily at early stages of embryonic development in central nervous system tissues. Their expression becomes nearly undetectable within a few days after birth [6]. GD3 and/or GD2 are frequently expressed in ectoderm-derived tumors (e.g. neuroblastoma, melanoma, T-cell leukemia, breast cancer) in adults, and are therefore considered to be cancer-associated antigens [7–9]. GD3, GD2, and certain other gangliosides are thought of as promising targets for cancer immunotherapy because their expression is restricted to malignant cells and they are accessible on the cell surface.

Both GD3 and GD2 activate signaling molecules, leading to distinctive phenotypes in different types of cancer cells. In clinical studies by M. Kaufmann's group, GD3 synthase (GD3S; glycosyltransferase encoded by the *ST8SIA1* gene) was more highly expressed in estrogen receptor (ER)-negative breast tumors, and had prognostic significance for ER status-dependent breast cancer [10, 11]. In a study of tumorigenesis mechanism, R.K. Yu's group found that GD3 colocalized and associated with epidermal growth factor receptor (EGFR, a mitogen receptor) in the microdomain structure of plasma membrane [12]. Such interaction preserved EGFR levels by employing an endosomal-plasma membrane recycling pathway following endocytosis of EGF. In this way GD3 facilitated EGF-mediated signaling and regulated cell-fate determination of neuronal stem cells. K. Furukawa's group observed high expression of GD3 in human melanoma and small cell lung cancer. Upregulation of GD3 promoted cell growth and invasion through integrin β1 assembly in lipid rafts, and mediated tyrosine phosphorylation of focal adhesion kinase, p130Cas, and paxillin [13, 14]. In studies by P. Delannoy's group, GD3S overexpression in breast cancer cell lines increased cell proliferation and migration in the absence of growth factors through activation of c-Met, PI3K/Akt, and mitogen-activated protein kinase (MAPK)/ERK pathways [15]. Colocalization of GD2 and c-Met was observed at the plasma membrane. Silencing of GM2/GD2 synthase significantly reduced GD2 expression and c-Met phosphorylation, and reversed the proliferative phenotype [16]. Together, these findings suggest that GD3S induction in breast cancer cells promotes tumor aggressiveness.

GD2 was identified as a specific cell surface marker of CD44hi/CD24lo breast cancer stem cells (CSCs) from human breast cancer cell lines and patient samples [17]. Reduction of GD2 expression by *ST8SIA1* knockdown inhibited mammosphere formation and cell motility, and completely blocked tumor formation *in vivo*, changing the CSC phenotype to a non-CSC phenotype [17, 18]. Induction of epithelial-mesenchymal transition (EMT) in transformed human mammary epithelial cells markedly increased expression of GD3S and GD2. Conversely, inhibition of GD3S prevented metastasis by interfering with initiation and maintenance of EMT [19]. GD3S expression is correlated with constitutive activation of the c-Met signaling pathway that leads to enhancement of stem cell properties and metastatic potential. The GD3S/c-Met axis may therefore be a useful target for treatment of metastatic breast cancer.

In a previous study, we compared expression levels of GSLs in breast CSCs vs. non-CSCs, and observed notably higher expression of GD2 and GD3 in breast CSCs [18]. Cell populations that expressed GD2/GD3 displayed a CD44^hi^/CD24^lo^ stem cell phenotype. Knockdown of the genes for GD2 synthase (*B4GALNT1*) and GD3S (*ST8SIA1*) significantly reduced GD2/GD3 expression and reversed the stem cell phenotype (e.g. mammosphere formation and enhanced motility) of breast cancer cells [18]. On the basis of these findings, we proposed a link between GD2/GD3 expression and maintenance of breast CSCs.

In the present study, we further investigated the effects of GD2 and GD3 on tumor-related phenotypes of breast CSCs. GD3S expression sustained CSC properties, including the upregulation of ALDH activity, formation of mammospheres, and expression of EMT markers. GD3S overexpression also promoted malignant properties such as cell migration, attachment, and colony formation. Transfection of GD3S cDNA into breast cancer cell lines MDA-MB231, MDA-MB468, and MCF7 cells resulted in cell type specific upregulation of GD2 and GD3. Furthermore, we found that GD3 colocalized and associated with EGFR, and it activated EGFR signaling in breast cancer cell lines and breast CSCs. In addition, GD3S knockdown in triple-negative MDA-MB468 cells resulted in increased sensitivity to gefitinib *in vitro* and *in vivo*. These findings clearly demonstrate the positive role of GD3S in maintaining CSC properties and in malignant progression. In light of the observed GD3/EGFR association in breast CSCs and breast cancer cell lines, along with the increased gefitinib sensitivity in GD3S-knockdown MDA-MB468 cells, we propose that GD3S is involved in gefitinib-resistance of EGFR-positive breast cancer cells, and can be considered as a potential therapeutic target in gefitinib-resistant breast cancers.

## RESULTS

### GD3S sustains stem cell phenotype of breast cancer cells

**Figure 1:**
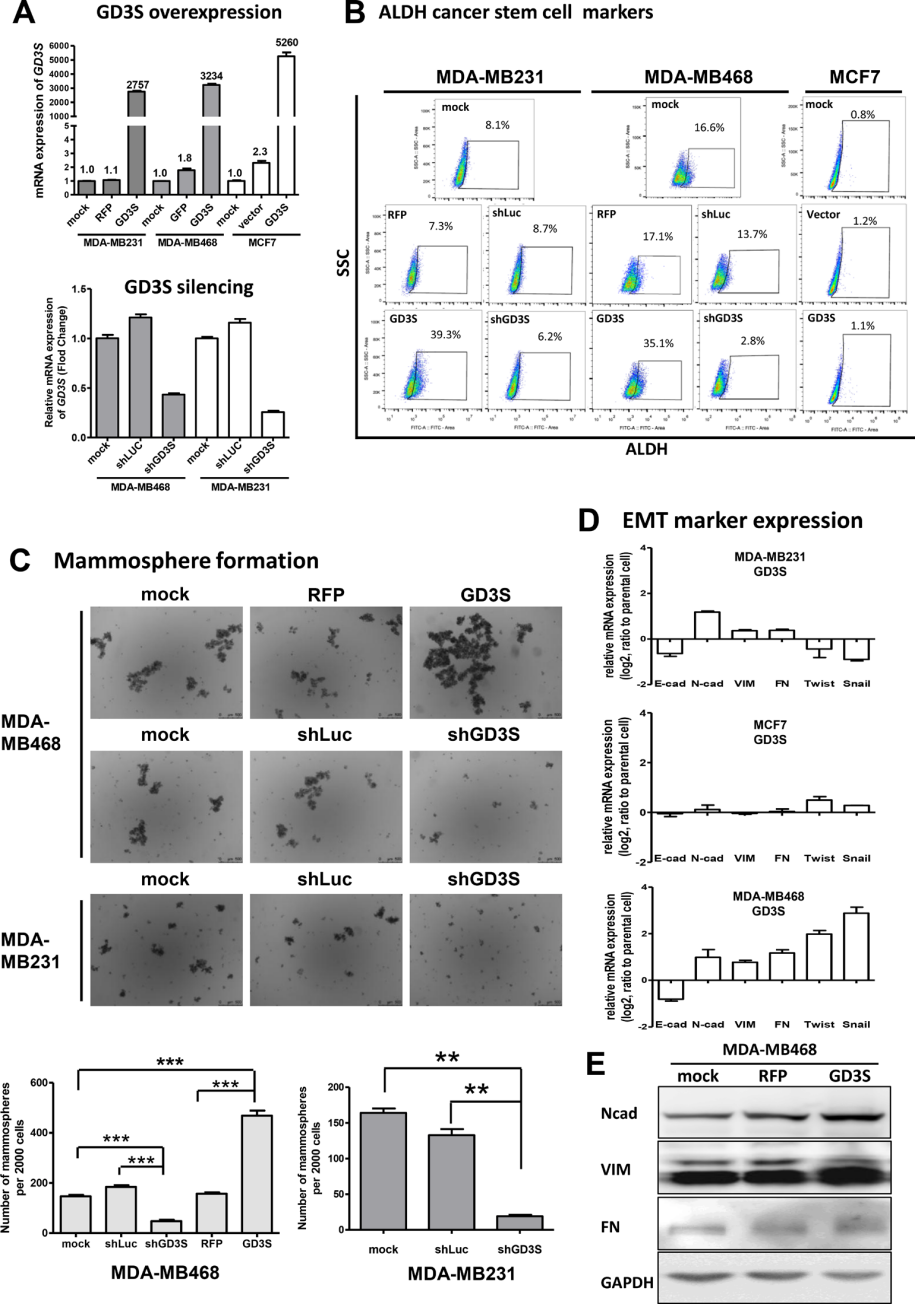
Effects of GD3 synthase (GD3S) gene expression on ALDH1 cancer stem cell (CSC) markers, epithelial-mesenchymal transition (EMT) markers, and mammosphere formation ability in three breast cancer cell lines Stable GD3S-overexpressing and -knockdown cell lines were established from MDA-MB231, MDA-MB468, and MCF7 cells. (**A**) GD3S mRNA expression assessed by real-time qRT-PCR. Numbers above bars: fold change of GD3S expression. (**B**) Flow cytometry analysis of ALDH1 activity in GD3S-overexpressing and control cell lines. Cells were suspended in ALDEFLUOR assay buffer containing BAAA substrate. Each sample was treated with DEAB as a negative control. Staining was measured with a SONY SA3800 spectral cell analyzer, and the proportion of ALDH1 bright cells is shown. (**C**) GD3S-overexpressing and -knockdown cells were plated onto ultralow attachment plates. Representative images of derived mammospheres are shown (magnification 20×). Number of spheres for each well was counted, and mammosphere formation efficiency was calculated as number of spheres formed per original number of cells seeded. Data are shown as mean ± SEM. (**D**) mRNA expression of EMT markers E-cadherin (E-cad), N-cadherin (N-cad), vimentin (VIM), fibronectin (FN), Twist, and Snail, assessed by real-time qRT-PCR. The experiments were performed in triplicate and repeated three times. Data are shown as mean ± SEM. (**E**) Immunoblot analysis of EMT markers, fibronectin (FN), vimentin (VIM) and N-cadherin (N-cad) in MDA-MB468 and MDA-MB468 cells with GD3S overexpression. GAPDH was used as loading control.

We demonstrated previously that GD3S, in combination with downstream GSL products (GD2 and GD3), plays a positive role in maintaining stem cell properties in human breast CSCs [18]. To facilitate more detailed studies of the biological roles of GD2 and GD3, we established breast cancer cell lines that stably overexpress GD3S by transfection of mammalian expression vector containing full-length cDNA of human GD3S into MDA-MB231, MDA-MB468, and MCF7 cells. GD3S mRNA was quantified by real-time qRT-PCR. In comparison with mock-transfected cells, red fluorescent protein (RFP)-tagged cells, or empty vector control cells, stable GD3S overexpression produced striking (2000- to 5000-fold) increases in GD3S mRNA levels in MDA-MB231, MDA-MB468, and MCF7 cells (Figure 1A, upper panel). The converse experiment of GD3S knockdown (*ST8SIA1* gene silencing) was performed using a lentiviral-based expression vector carrying shGD3S. In this case, real-time qRT-PCR indicated that GD3S expression level was reduced by approximately 70% in MDA-MB468 and roughly 60% in MDA-MB231 cells (Figure 1A, lower panel).

Aldehyde dehydrogenase isoform 1 (ALDH1) activity is a metabolic feature commonly used for identification and analysis of CSC progression [20]. To assess the contribution of GD3S to CSC properties, we evaluated ALDH1 activity in GD3S-overexpressing and -knockdown breast cancer cell lines. Presence and size of cell populations showing measurable ALDH enzymatic activity were determined by the ALDEFLUOR assay with flow cytometry analysis (Figure 1B). Cells incubated with ALDH1 substrate BAAA or ALDH1 inhibitor DEAB were used to establish baseline fluorescence values and to define ALDH1-positive populations. In GD3S-overexpressing MDA-MB231, the percentage of ALDH1-positive cell population was significantly upregulated, from 8.1% to 39.3%. In GD3S-overexpressing MDA-MB468 cells, the ALDH1-positive percentage increased from 16.6% to 35.1%. In MCF7 cells, GD3S overexpression had no significant effect on ALDH1 activity. GD3S knockdown by shGD3S caused a significant reduction in ALDH1-positive percentage (from 16.6% to 2.8%) in MDA-MB468 cells, but had no effect on ALDH1 activity in MDA-MB231 cells (Figure 1B).

For MDA-MB468 cells, mammosphere formation ability was approximately 3-fold higher in GD3S-overexpressing cells than in parental controls, but was reduced to 26% of parental control levels in GD3S-knockdown cells (Figure 1C). GD3S knockdown reduced mammosphere formation to 15% in MDA-MB231 cells (Figure 1C). GD3S overexpression in MCF7 cells had no effect on mammosphere formation ability (data not shown).

Epithelial-mesenchymal transition (EMT) is an important mechanism in cancer metastasis, and is involved in acquisition and maintenance of stem cell-like characteristics [21]. GD3S-overexpressing MCF7, MDA-MB231, and MDA-MB468 cell lines showed evidence of EMT, including *E-cadherin* downregulation and *N-cadherin* upregulation (Figure 1D). In addition, GD3S-overexpressing MDA-MB468 cells displayed upregulation of *vimentin*, *fibronectin*, *Twist*, and *Snail*, which are also indicators of EMT (Figure 1D). GD3S-mediated upregulation of N-cadherin, vimentin and fibronectin, three of the main markers of EMT, was confirmed at the protein level by immunoblot (Figure 1E).

Taking these data together, the ALDEFLUOR assay, mammosphere formation assay, and analysis of EMT markers expression revealed differential effects of GD3S overexpression on stem cell properties of the three cell lines. In MDA-MB231 and MDA-MB468, which are triple-negative breast cancer (ER, PR, and HER2 negative; TNBC) cell lines, GD3S overexpression enhanced stem cell properties. In contrast, GD3S overexpression had no effect on stem cell properties of MCF7, which is of luminal type and ER and PR positive.

### GD3S enhances migration, adhesion, and clonogenic growth of breast cancer cells

**Figure 2:**
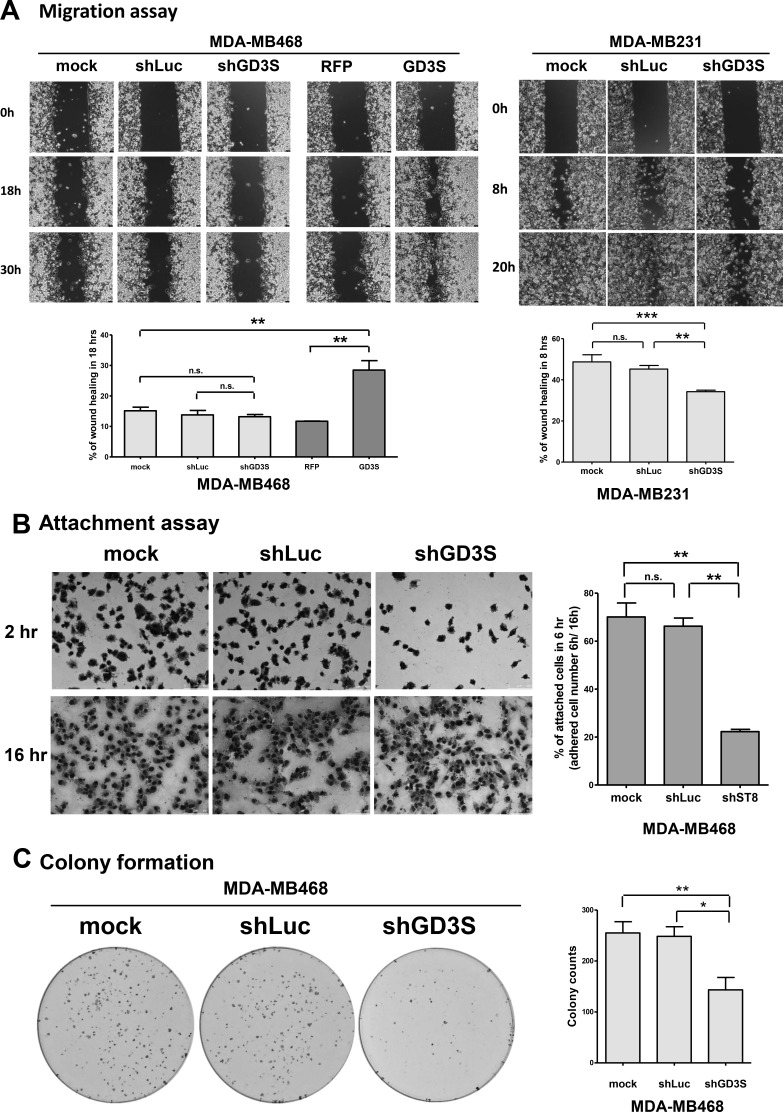
Effects of GD3S expression on cell migration, attachment efficiency, and clonogenic growth potential (**A**) GD3S-overexpressing and -knockdown cell lines were tested with a wound healing assay. A clean wound area was scratched with a pipette tip, and wound healing movement was monitored by live-cell time-lapse microscopy. Images are shown at 0, 18, and 30 h for MDA-MB468 cells, and at 0, 8, and 20 h for MDA-MB231 cells. Wound healing area percentages at 18 h (MDA-MB468) and 8 h (MDA-MB231) was quantified using ImageJ software. (**B**) Cell attachment assay. MDA-MB468 cells *expressing ST8SIA1*-targeting shRNA (shGD3S) or non-targeting control (shLuc) were seeded for 6 h to 50-75% confluency in culture medium with 10% FBS. Wells were washed with PBS to remove non-adherent cells, and remaining adherent cells were fixed and stained by crystal violet. Percentage of attached cells was calculated as (adherent cell number remaining at 6 h/adherent cell number remaining at 16 h) × 100%. (**C**) Clonogenic growth potential assay. Three types of MDA-MB468 cells as in (B) were seeded on 6-well plates (*n* = 600) and incubated 14 d. Colonies were stained with crystal violet, measured, and values were expressed as mean ± SEM (n = 3). *p < 0.05; **p < 0.01; ***p < 0.001; n.s. = not significant.

We further investigated the role of GD3S expression in tumor phenotypes of TNBC cell lines, MDA-MB231 and MDA-MB468. Mechanisms that regulate wound healing have been suggested to promote transformation and growth of malignant cells. Moreover, wound healing-related inflammatory cytokines and growth factors have been identified as key contributors to the CSC niche [22]. We therefore examined how GD3S expression influences cell migration using wound healing (scratch) assays. In Figure 2A, GD3S-overexpressing MDA-MB468 cells displayed a 2-fold increase in wound healing efficiency in comparison with control RFP-tagged vector cells (left panel). GD3S-knockdown in MDA-MB468 cells showed no change in migration potential relative to control shLuc cells (left panel). In contrast, GD3S-knockdown in MDA-MB231 cells showed a significant reduction of migration potential (from 50% to 36%) relative to shLuc cells (Figure 2A, right panel). These findings demonstrate that GD3S overexpression enhances cell migration. Because GD3S promoted EMT in MDA-MB231 and MDA-MB468 cells (Figure 1D), enhanced migration may be attributed to the acquisition of an EMT-derived mesenchymal-like phenotype.

Cell attachment and detachment properties in the tumor microenvironment are determinants of cell migration and invasion during metastatic processes [13]. We examined the effect of GD3S knockdown on attachment efficiency in MDA-MB468 cells. Two hours after seeding, non-adherent cells were washed away, and adherent cells were visualized by crystal violet staining. The percentage of adherent cells was significantly decreased (from 70% to 22%) in knockdown cells relative to control cells (Figure 2B). Thus, attachment capacity was reduced by GD3S knockdown, consistent with the reduction of migration potential.

The colony formation assay (clonogenic assay) is widely used for quantification of transforming potential and for assessing the tumorigenic nature of cells [23]. Clonogenic growth potential of GD3S-knockdown MDA-MB468 cells was significantly reduced (by 46.2%) relative to mock-transduced controls (Figure 2C). This finding suggests that GD3S expression is involved in regulation of clonogenic growth potential, and may therefore be correlated with clinical outcomes such as tumor regrowth and disease relapse.

Our assays measuring CSC and cancer cell properties (Figures 1 and 2) indicate that GD3S has an oncogenic function in mammary epithelial cell tumorigenesis. This oncogenic function includes maintaining stem cell-like features and promoting cell migration and transformation, particularly in TNBC and basal-like breast cancer cells such as MDA-MB231 and MDA-MB468 cell lines.

### Cell type-specific regulation of GD2/GD3 biosynthesis by GD3S expression

**Figure 3:**
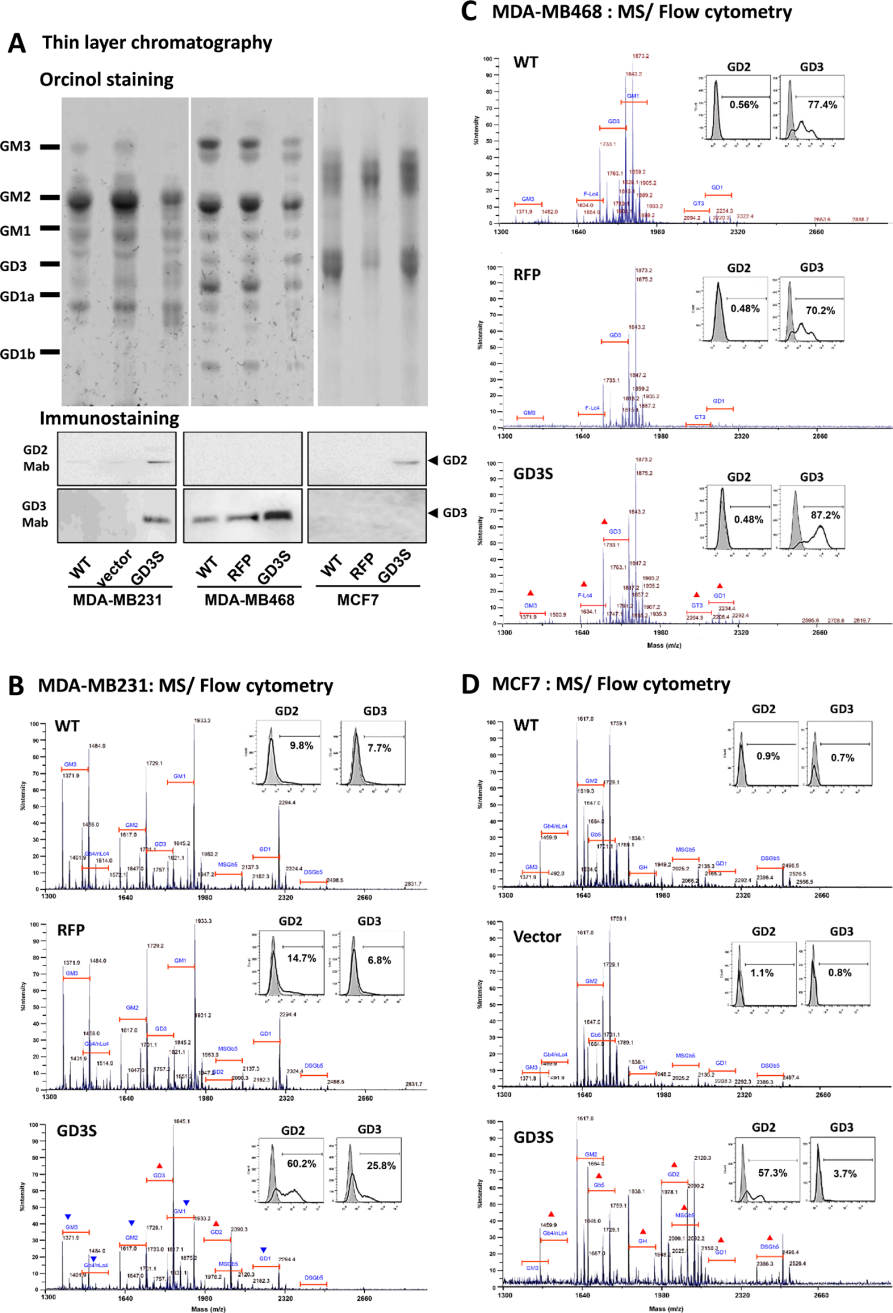
GD3S expression modifies GSL profiles in breast cancer cell lines (**A**) Upper-phase GSLs were separated on HPTLC plates with solvent system chloroform/ methanol/ 0.2% CaCl_2_ in H_2_O (50:40:10, v/v/v), and visualized by orcinol spray (upper panel). GSL extract from each cell line was immune-stained with anti-GD2 (middle panel) or anti-GD3 (lower panel) mAb on TLC plate. Intact GSLs from MDA-MB231 (**B**), MDA-MB468 (**C**), and MCF7 (**D**) were permethylated and analyzed by MALDI-TOF MS. Parental untransfected wildtype control (WT), RFP/vector control, and GD3S-overexpressing transfectants are shown in parallel. Values shown are m/z of sodium adducted [M + Na]^+^ molecular ions. Annotations of GSLs on spectra were assigned based on m/z values typical for ceramide moiety-associated fatty acyl heterogeneity. GSLs having the same glycan moiety but different fatty acyl components are bracketed as a group. Red triangles (▲): upregulated expression of GSLs. Blue inverted triangles (▼): downregulated expression of GSLs. GD2 and GD3 expression for each cell line was analyzed by flow cytometry using specific mAbs. Gray histograms: cells treated with isotype control. Bold black lines: fluorescence-positive cells. Percentages of number of cells having fluorescence values > 95% of isotype control value are shown.

GD3S expression leads to distinctive phenotypes in different breast cancer cell lines. We examined the effect of GD3S expression on GSL biosynthesis by measuring GSL expression in GD3S-overexpressing MDA-MB231, MDA-MB468, and MCF7 cells. Total GSLs were extracted, analyzed by HPTLC, and visualized with orcinol spray (Figure 3A, upper panel). The three cell lines showed distinctive GSL expression patterns. GD2 and GD3 expression was confirmed by TLC immunostaining with anti-GD2 mAb (14G2a) and anti-GD3 mAb (R24) (Figure 3A, lower panels). GD2 was upregulated in MCF7 and MDA-MB231 cells, while GD3 was upregulated in MDA-MB231 and MDA-MB468 cells. GD3S-overexpressing MDA-MB468 cells showed enhanced GD3 expression (with parental cells already GD3-positive), but no detectable GD2 expression. In contrast, GD3S-overexpressing MCF7 cells showed enhanced expression of GD2 but not of GD3. Thus, GD3S overexpression had differential effects on GD2 and GD3 expression in the three cell lines. More specifically, GD3S overexpression resulted in expression of both GD2 and GD3 expression in MDA-MB231 cells, GD3 expression in MDA-MB468 cells, and GD2 expression in MCF7 cells.

Furthermore, differential GD2 and GD3 expression patterns on the cell surface were confirmed by flow cytometry analysis. The three cell lines were labeled by anti-GD2 mAb (14G2a) or anti-GD3 mAb (R24) conjugated with fluorophore. In representative histograms (Figure 3B–3D, insets), gray peaks correspond to isotype control and black lines correspond to ganglioside mAb-specific fluorescent signals. Flow cytometry analysis was consistent with results of TLC immunostaining, and the results demonstrate distinct, cell-type specific effects of GD3S overexpression on GD2 and GD3 expression patterns. Upregulated expression of both GD2 and GD3 were found in MDA-MB231 cells. GD3 (but not GD2) was upregulated in MDA-MB231 cells, and GD2 (but not GD3) was upregulated in MCF7 cells.

To evaluate changes of GSL profiles in the GD3S-overexpressing cell lines, purified GSLs were also analyzed by MALDI-TOF MS (Figure 3B–3D). Identities of GSLs in MS1 profiles were assigned based on m/z values for major molecular ion signals, fitted to permethylation of hexose, N-acetylhexosamine, deoxyhexose, and N-acetylneuraminic acid residues, in combination with typical sphingosine and fatty acyl components of common ceramides. GSL structures were inferred on the basis of known human GSL expression patterns.

The major GSLs expressed by wild-type (WT) MDA-MB231 cells and RFP-tagged control cells were a-series gangliosides (GM1, GM2, and GM3). The b-series gangliosides (GD1, GD3) and globo-series GSLs (Gb4, SSEA4 [monosialoGb5; MSGb5], disialoGb5 [DSGb5]) were detected at low levels. Relative to un-transfected WT or RFP-tagged control cells, GD3S overexpression resulted in upregulation of molecular ion signals corresponding to GD2 and GD3, and downregulation of signals corresponding to GM3, GM2, GM1, GD1, and Gb4/(n)Lc4 (Figure 3B).

As shown in Figure 3C, WT MDA-MB468 cells expressed predominantly GM1 and GD3, and low levels of GM3, GT3, and GD1. Fucosyl-Lc4 were also detected at low levels. GD3S overexpression in these cells resulted in upregulation of signals corresponding to GD3 (not GD2), and, to a lesser degree, increase of complex gangliosides GT3 and GD1.

In WT MCF7 cells, the only highly expressed ganglioside was GM2. These cells expressed mainly neutral GSLs (Gb4, Gb5, and globo-H), and lower levels of sialylated GSLs (GM3, MSGb5, and DSGb5). GD3S overexpression led to upregulation of signals corresponding to GD2, but not GD3 (Figure 3D). MS analysis revealed increases of various globo- and lacto-series GSLs (Gb4/(n)Lc4, Gb5, globo-H, MSGb5, and DSGb5) and to a lesser degree, complex ganglioside GD1. In summary, findings shown in Figure 3 clearly demonstrate the differential effects of GD3S overexpression on GD2/GD3 expression patterns in the three breast cancer cell lines.

### Interaction of gangliosides and GFRs in breast CSCs

**Figure 4:**
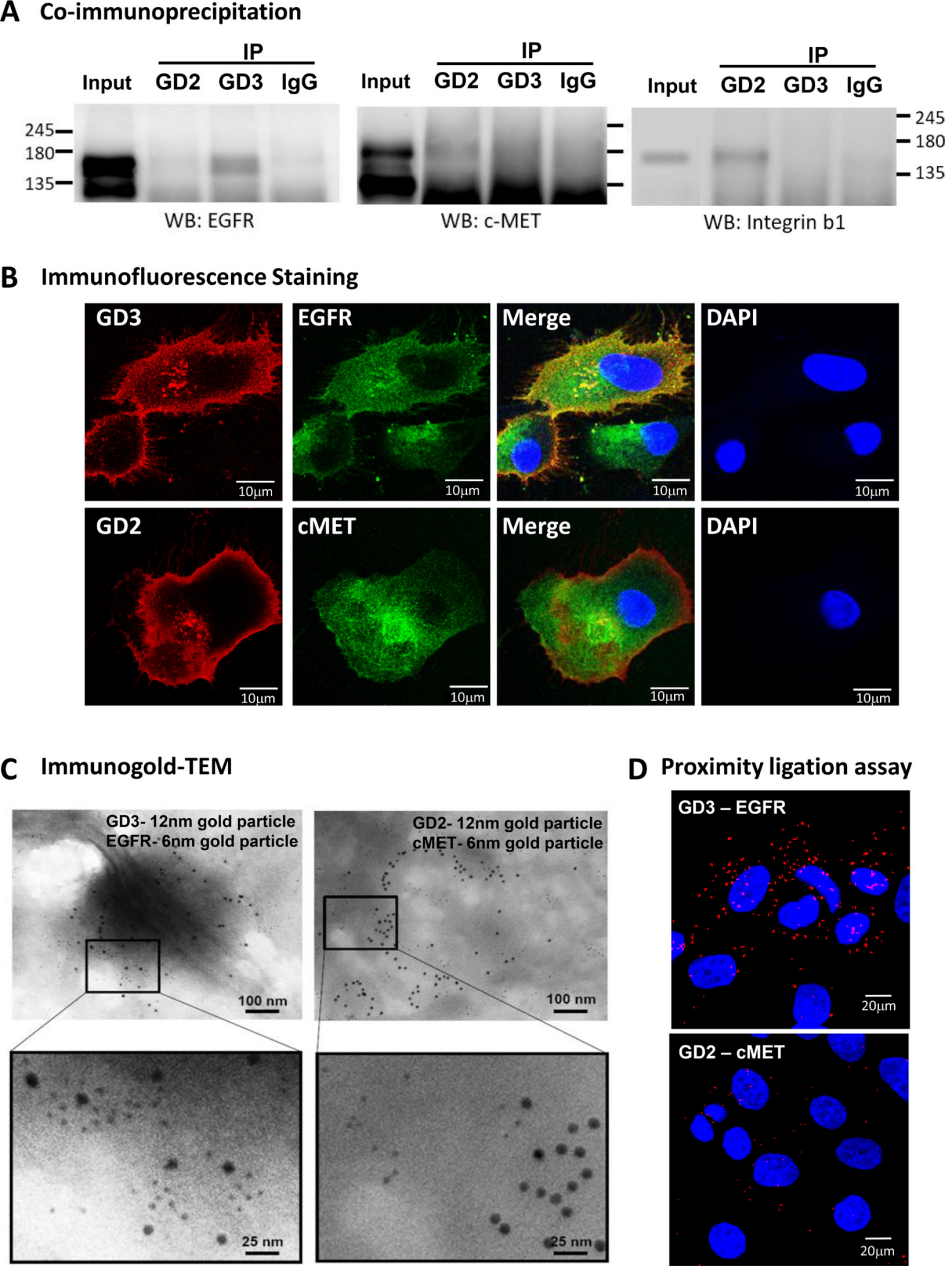
Association of GD2 and GD3 with GFRs in breast CSCs Association between GD2/GD3 and GFRs was evaluated using Twist-induced EMT of HMLE-Twist-ER cells (breast CSCs). (**A**) Cell lysates of breast CSCs were immunoprecipitated (IP) with mouse anti-GD2 mAb, anti-GD3 mAb, or normal mouse IgG, followed by Western blotting (WB). WB was probed with anti-EGFR (left panel), anti-c-Met (middle panel), or anti-integrin β1 (right panel) mAb to detect components of Ab-absorbed complexes. Integrin β1/GD2 association was used as positive control. (**B**) Semi-confluent monolayers of breast CSCs were fixed and permeabilized. Cells were immune-stained with anti-GD3 (red) and anti-EGFR (green) (upper panels) or with anti-GD2 (red) and anti-c-Met (green) (lower panels), for immunofluorescence double labeling in combination with DAPI staining (blue) for cell nuclei. Scale bar = 10 μm. (**C**) GD3/EGFR and GD2/c-Met associations were evaluated by immunogold-TEM. Gangliosides (GD2, GD3) were probed with 12 nm colloidal gold particles; GFRs (EGFR, c-Met) were probed with 6 nm colloidal gold particles. Scale bar = 100 or 25 nm. Regions indicated by white boxes are shown at higher magnification in lower panels. Scale bar = 100 or 25 nm. (**D**) GD3/EGFR and GD2/c-Met associations in breast CSCs were investigated by *in situ* proximity ligation assay (PLA). Each PLA signal is visualized as a red fluorescent spot, and represents one detected association event. Cell nuclei were stained with DAPI (blue). Scale bar = 20 μm.

Our 2013 study [18] indicated that GD2/GD3 and their upstream glycosyltransferases, GD2/GM2 synthase and GD3S, were upregulated in breast CSCs. Knockdown of these enzymes reversed CSC properties, suggesting the involvement of GD2/GD3 in maintenance of these properties. In the present study, we evaluated the potential for association between GFRs and gangliosides. This was done to clarify the mechanism whereby association of membrane protein molecules with GD2/GD3 maintains stem cell properties of breast CSCs. Such associations were first investigated by co-immunoprecipitation (co-IP) experiments. Cell lysates from breast CSCs were immunoprecipitated with anti-GD2 or anti-GD3 mAb and then immunoblotted with anti-EGFR or anti-c-Met mAb. GD3 associated with EGFR (Figure 4A, left panel), but not with c-Met (Figure 4A, middle panel). GD2 associated less obviously with c-Met (Figure 4A, middle panel), but not with EGFR (Figure 4A, left panel). Consistent with a previous report [24], integrin β1 associated with GD2, but not with GD3 (Figure 4A, right panel).

Such ganglioside/GFR associations in breast CSCs were further investigated by immunostaining. GD3 (red) and EGFR (green) showed a high degree of colocalization (yellow) (Figure 4B, upper panels). Consistent with co-IP results, GD2 (red) and c-Met (green) showed only partial colocalization (yellow) (Figure 4B, lower panels).

Intracellular colocalization of gangliosides and GFRs was evaluated by immunogold-TEM (Figure 4C). Using two secondary Abs conjugated to gold particles of different sizes, two different antigens (in this case, ganglioside and GFR) can be visualized simultaneously by TEM. Breast CSCs were double-labeled with 12 nm (for gangliosides) and 6 nm (for GFRs) colloidal gold particles. The distributions of differently-sized gold particles indicate preferential colocalization of GD3 (12 nm particles) and EGFR (6 nm particles) (Figure 4C, left panel). In contrast, GD2 (12 nm particles) was mostly separated from c-Met (6 nm particles) (right panel).

*In situ* interactions of gangliosides and GFRs were investigated by a proximity ligation assay (PLA). In this technique, when a pair of PLA probes binds two molecules that are in close proximity (< 16 nm), complementary DNA strands conjugated to PLA probes are ligated, amplified, and visualized as distinct points using a fluorescent probe. In breast stem-like cancer cells, strong PLA signals were observed for GD3/EGFR association, whereas less obvious PLA signals were observed for GD2/c-Met association (Figure 4D). Taken together, the results in Figure 4 clearly demonstrate a novel GD3/EGFR association in EMT-induced breast stem-like cancer cells.

### GD3/EGFR and GD2/c-Met association in GD3S-overexpressing breast cancer cell lines

**Figure 5:**
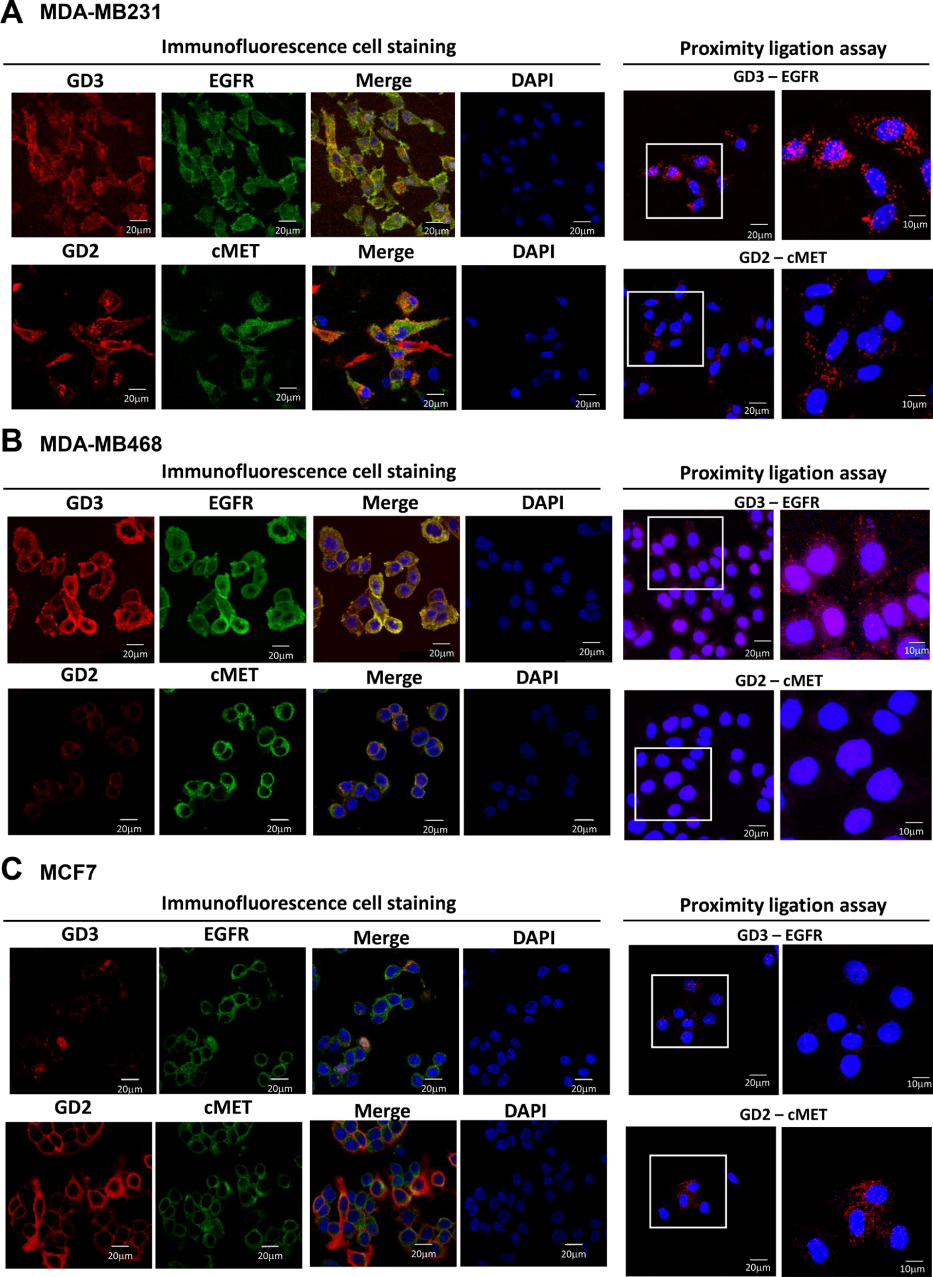
Colocalization of GD2 and GD3 with GFRs in GD3S-overexpressing breast cancer cell lines (**A**–**C**) GD3S-overexpressing MDA-MB231, MDA-MB468, and MCF7 cells were fixed, permeabilized, and then stained for immunofluorescence (left images) by anti-GD3 (red)/ anti-EGFR (green) or anti-GD2 (red)/ anti-c-Met (green). Nuclei were stained with DAPI (blue). Colocalization signals are shown as yellow in merged images. GD3/EGFR and GD2/c-Met associations were further investigated by *in situ* PLA (right images). Each PLA signal is visualized as a red fluorescent spot, and represents one detected association event. Regions indicated by white boxes are shown at higher magnification in lower panels. Scale bar = 20 or 40 μm.

To investigate interactions between gangliosides and GFRs, distributions of GD2, GD3, EGFR, and c-Met in the same three cell lines were analyzed by immunofluorescence staining and *in situ* PLA. In MDA-MB231 cells, both GD2 and GD3 were expressed, and GD2 colocalized with c-Met (Figure 5A), in agreement with previous reports [15, 16]. In GD3S-overexpressing MDA-MB231 cells, GD3 colocalized with EGFR (Figure 5A, left panels). *In situ* PLA also demonstrated GD3/EGFR and GD2/c-Met colocalization in GD3S-overexpressing MDA-MB231 cells (Figure 5A, right panels). In GD3S-overexpressing MDA-MB468 cells, which express high levels of GD3 but no GD2, GD3/EGFR colocalization and association were demonstrated by immunofluorescence staining (Figure 5B, left panels) and *in situ* PLA (Figure 5B, right panel). GD3S-overexpressing MCF7 cells express high GD2 but no GD3, and therefore showed no GD3/EGFR colocalization signal. Despite the high GD2 expression in these cells, both immunofluorescence staining and *in situ* PLA revealed limited GD2/c-Met colocalization or association (Figure 5C). Taken together, the results from Figure 5 demonstrate an association between GD3 and EGFR in GD3S-overexpressing breast cancer cell lines, consistent with findings from EMT-induced breast CSCs shown in Figure 4.

### GD3S expression enhances EGFR signaling in breast CSCs and breast cancer cell lines

**Figure 6:**
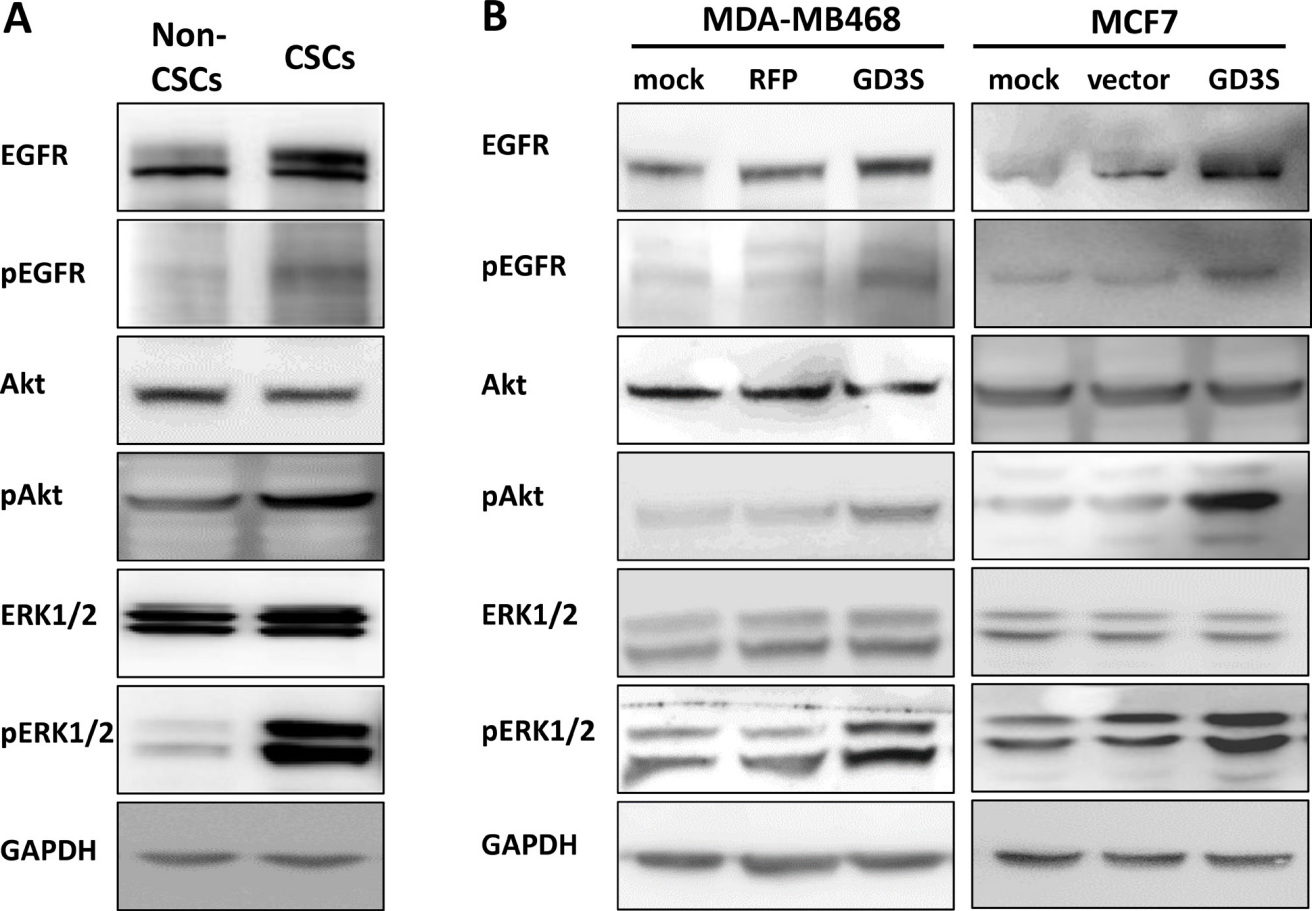
Activation of EGFR signaling pathways in breast CSCs and cancer cell lines with high GD3S expression (**A**) Cell lysates (50 mg) of breast non-CSCs and CSCs were analyzed by immunoblotting with mAbs directed against the indicated EGFR signaling molecules. Breast CSCs with high GD3S expression showed increased EGFR, p-EGFR, p-AKT, and p-ERK1/2 signaling, with GAPDH used as loading control. (**B**) Breast cancer cell lines MDA-MB468 and MCF7 were transduced with lentivirus carrying GD3S cDNA (GD3S) or vector control plasmid (vector). Cell lysates (50 μg) were immunoblotted with mAbs directed against the same EGFR signaling molecules as in panel A. GD3S-overexpressing cell lines showed increased EGFR, p-EGFR, p-AKT, and p-ERK1/2 signaling, with GAPDH as loading control.

To investigate the effects of GD3S expression on EGFR signaling pathways, we examined EGFR protein levels and downstream signaling in GD3S-expressing breast CSCs and breast cancer cell lines by immunoblotting. In comparison with non-CSCs, breast CSCs with high GD3S expression showed increased levels of EGFR protein, phosphorylated EGFR (p-EGFR), AKT (p-AKT), and p44/42 MAPK (p-ERK1/2) (Figure 6A, left panel). Effects of GD3S overexpression on EGFR signaling pathways in MDA-MB468 and MCF7 cells were also examined. EGFR, p-EGFR, p-AKT, and p-ERK1/2 levels were also upregulated in the GD3S-overexpressing cells (Figure 6B). These findings suggest that GD3S enhances CSC properties and tumor phenotypes of breast cancer cells through EGFR signaling.

### GD3S knockdown enhances sensitivity of TNBC MDA-MB468 cells to gefitinib

**Figure 7:**
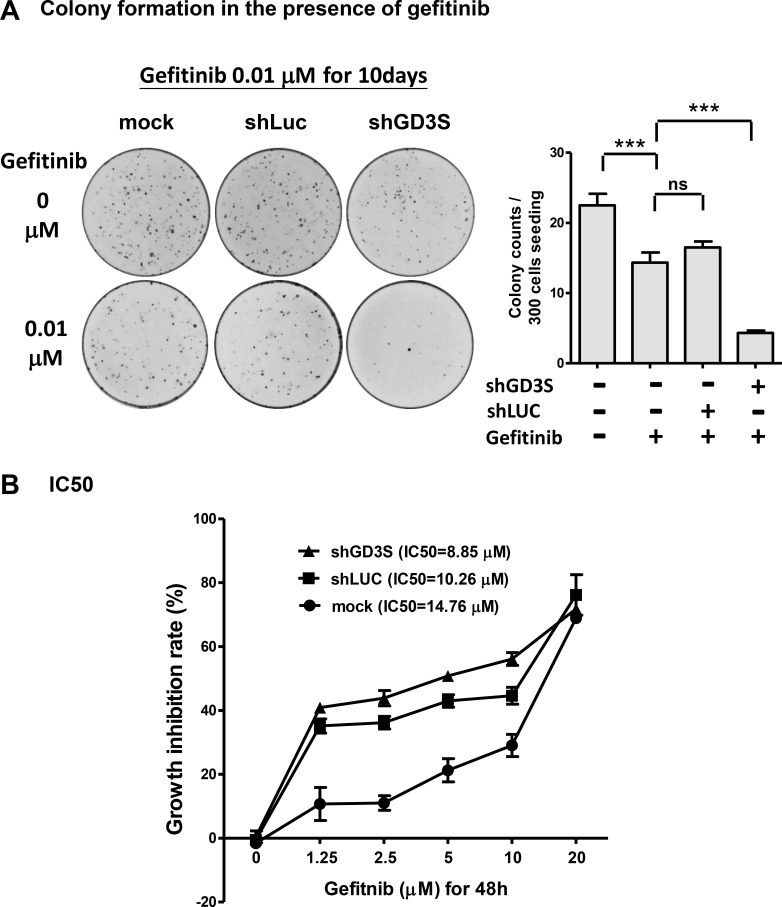
GD3S knockdown sensitizes breast cancer MDA-MB468 cells to EGFR kinase inhibitor gefitinib (**A**) Reduction of clonogenic growth potential by combination GD3S knockdown (by shGD3S) and gefitinib treatment. Cells (*n* = 500) were seeded on 3.5-cm dishes, incubated 16 h, and treated with 0.01 μM gefitinib for 10 d. Colonies were stained with crystal violet (upper panel) and measured. Lower panel: colony counts. **p < 0.01; ***p < 0.001; ns = not significant. (**B**) MDA-MB468 cells with or without GD3S knockdown were treated with serial concentrations (0, 1.25, 2.5, 5, 10, 20 μM) of gefitinib for 48 h, and gefitinib IC50 value was determined by alamarBlue assay. The data were analyzed by one-way ANOVA followed by Fisher's least significant difference method. The difference of growth inhibition rate between GD3S-knockdown and mock control at gefitinib concentration of 2.5, 5 and 10 μM were statistically significant (*p* < 0.05).

Gefitinib, a therapeutic for certain types of breast, lung, and other cancers, is a tyrosine kinase inhibitor that targets the ATP binding site in the cytoplasmic domain of EGFR [25]. Most clinical studies have shown limited efficacy of gefitinib against breast cancer [26]. In view of our findings that GD3 associates with EGFR in breast stem-like cancer cells (Figure 4) and in GD3S-overexpressing breast cancer cell lines (Figure 5), and that GD3S expression enhances malignant potential of breast cancer cell lines (Figure 1 and 2), we hypothesized that a combination of gefitinib treatment and GD3S knockdown may be an effective therapeutic strategy to combat breast cancer. MDA-MB468 cells were treated for 10 d with 0.01 μM gefitinib in combination with GD3S knockdown (by shGD3S), after which clonogenic potential and cytotoxicity were evaluated (Figure 7). In comparison with parental or control shLuc cells, GD3S knockdown greatly enhanced growth inhibition by gefitinib (Figure 7A), and reduced the gefitinib IC50 value, after 48 h treatment, from 14.76 to 8.85 μM (Figure 7B). The differences between growth inhibition rates of GD3S knockdown and mock controls, at gefitinib concentrations of 2.5, 5, and 10 μM were statistically significant (*p* < 0.05). Thus, GD3S knockdown sensitized breast cancer cells to the EGFR kinase inhibitor gefitinib. The GD3S-knockdown-mediated enhancement of gefitinib cytotoxicity and reduction of gefitinib IC50 in TNBC cells (MDA-MB468) suggests that GD3S regulation is a useful target for breast cancer therapy.

### Gefitinib treatment in combination with GD3S knockdown enhances suppression of tumor growth *in vivo*

**Figure 8:**
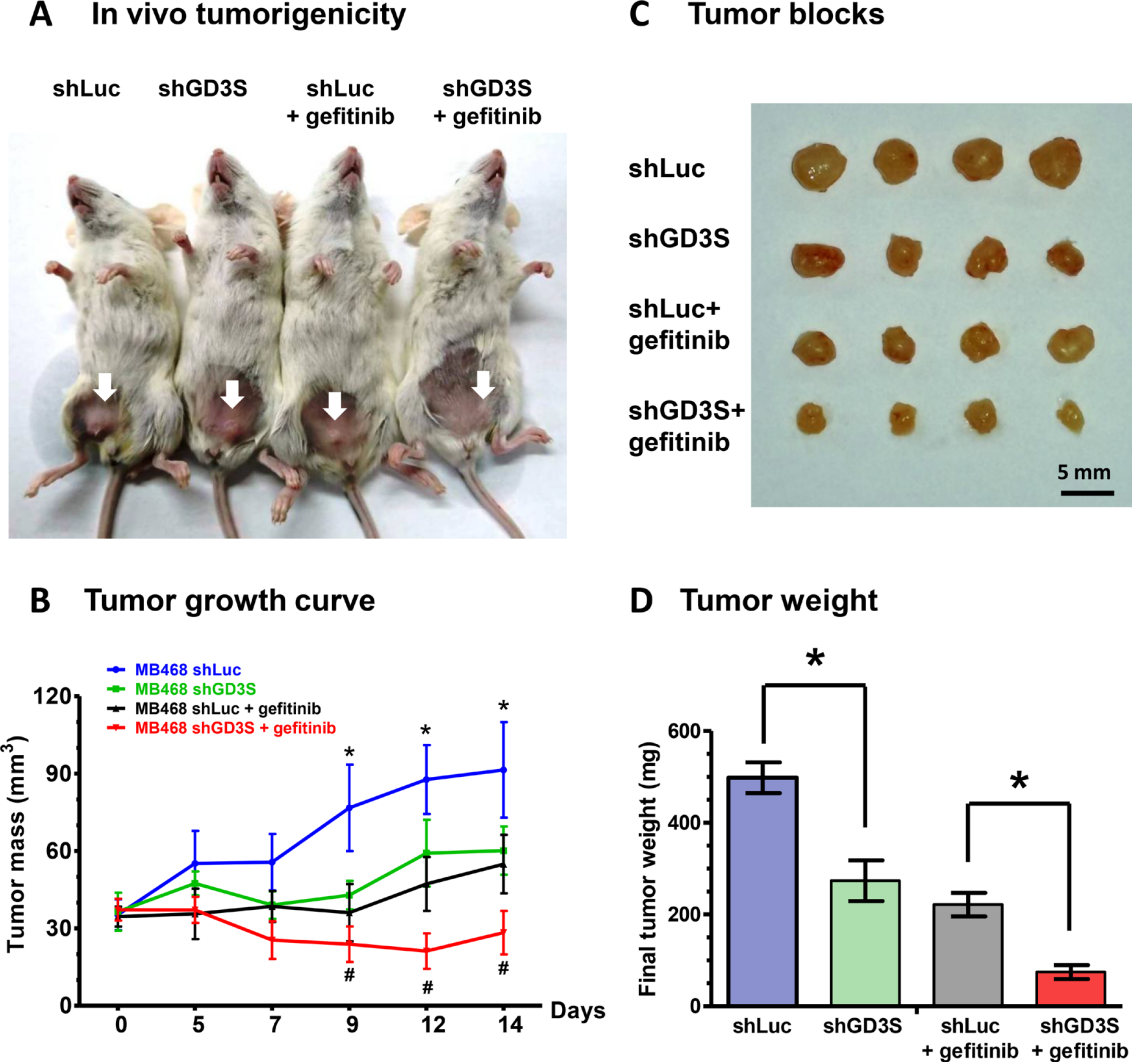
GD3S knockdown enhances the tumor-suppressive effect of gefitinib in mouse xenograft models (**A**) NOD/SCID/IL2Rgamma null (NSG) mice bearing xenografts of MDA-MB468-shLuc and MDA-MB468-shGD3S cells were treated with gefitinib (150 mg/kg) or PBS by oral gavage twice weekly for 16 d. Mice were then sacrificed, and tumors were dissected. (**B**) Tumor volume changes were recorded three times weekly, beginning on day 1 of gefitinib treatment. Data are shown as mean ± SD. *significant difference (*P <* 0.05) between means of MDA-MB468-shLuc and MDA-MB468-shGD3S groups. ^#^significant difference (*P <* 0.05) between means of gefitinib-treated MDA-MB468-shLuc and MDA-MB468-shGD3S groups. (**C**) Representative photographs of four tumor blocks from each of the four experimental groups on day 16. (**D**) Tumors were weighed on day 16. Data are shown as mean ± SD. *significant difference (*P <* 0.05).

We further evaluated the effect of GD3S on gefitinib resistance using xenograft breast cancer mouse models. NSG mice were injected via mammary fat pad with MDA-MB468 cells transduced with lentivirus carrying control or GD3S shRNA, to induce formation of tumors. Mice were subsequently treated with gefitinib (dose 150 mg/kg) by oral gavage twice weekly for 16 d. In comparison with the group treated with gefitinib alone, the group treated with gefitinib in combination with GD3S knockdown showed significantly greater suppression of tumor growth (Figure 8A and 8B). Tumor volume and tumor weight were also much lower in the gefitinib + GD3S knockdown group than in the gefitinib-only group (Figure 8C and 8D). These findings, taken together, indicate that GD3S inhibits TNBC cell growth and enhances the tumor-suppressive effect of gefitinib *in vitro* and *in vivo*.

## DISCUSSION

In our 2013 study [18], we compared GSL expression profiles in breast non-CSCs and CSCs using the EMT model developed by Weinberg's group. We found that CSCs had greatly reduced levels of Fuc(n)Lc4Cer and Gb3Cer, and much higher levels of GD2, GD3, GM2, and GD1a. Cell populations expressing GD2 and GD3 displayed a CD44^hi^/CD24^lo^ stem cell phenotype. Knockdown of the gene encoding GD3S (*ST8SIA1*) reduced both GD2 and GD3 expression, while knockdown of the gene encoding GD2 synthase (*B4GALNT1*) only reduced GD2 expression. Silencing either of these two genes reversed the stem cell phenotype (e.g. mammosphere formation and enhanced motility) of breast cancer cells. However, silencing of *ST8SIA1* showed the most potent effect on suppression of stem cell properties. These findings demonstrated a positive functional role for GD2 and GD3 in breast CSCs and suggested a possible approach to inhibiting tumorigenic breast CSCs by targeting the GD2/GD3 synthase-related pathway. In the present study, we performed a more detailed examination of the biological impact of GD2 and GD3 in driving a stem cell phenotype and enhancing tumorigenicity.

Breast cancer is a highly heterogeneous disease, in terms of both pathology and molecular profiles. Various molecular subtypes of breast cancer differ substantially with regard to diagnosis, prognosis, and treatment [27, 28]. We therefore used three breast cancer cell lines classified as different subtypes to investigate the functional effects of GD3S. These cell lines were selected to represent luminal-like and basal-like breast cancer subtypes, according to gene expression studies [29–33]. MCF7 is a luminal-like cell line that is ER+ PR+ HER2-, while MDA-MB231 and MDA-MB468 are both TNBC, based on the lack of ER, PR, and HER2. MDA-MB231 displays basal-like properties and is classified as claudin-low with enhanced invasive properties. MDA-MB468 is basal-like with PTEN deletion and EGFR amplification. After establishing stable GD3S-overexpressing cell lines, we used pooled populations of antibiotic-selected cells rather than isolated specific clones for biological assays and GSL analysis. This was done to avoid clonal variation.

In studies of the GD3S-overexpressing cell lines, we observed enhanced EMT and ALDH expression in basal-like TNBC cells, but not in luminal-type cells. This finding is consistent with previous reports that GD3S expression is necessary for maintenance of CSC properties in EMT-induced human mammary epithelial cells [17, 18]. S. Mani's group showed that inhibition of GD3S by shRNA or triptolide prevented EMT, blocked metastasis, and depleted stem cell populations [19]. By analyzing microarray data from 1,581 breast cancer samples, M. Kaufmann's group showed that GD3S expression was correlated with poor histopathological grade (*p* < 0.001) [10, 11]. These previous and our current findings indicate that GD3S is overexpressed in aggressive breast cancers and plays a positive role in regulation of EMT and stem cell function. Furthermore, the findings suggest a novel therapeutic strategy to combat aggressive breast cancers, which particularly targets therapy-resistant CSCs.

GSLs have been proposed to interact with specific membrane molecules to modify signaling transduction through carbohydrate-carbohydrate or protein-carbohydrate interactions [34]. We used HPTLC, flow cytometry, and MALDI-TOF MS analysis to establish comprehensive GSL profiles of our three breast cancer cell lines and their GD3S-overexpressing counterparts, and determine how GD3S expression alters expression of individual GSLs, affecting cancer cell and CSC phenotypes. GD3S catalyzes transfer of sialic acid from CMP-sialic acid to GM3, to produce gangliosides GD3 and GT3. GD3S is therefore the key enzyme for biosynthesis of b- and c-series gangliosides. We observed distinct GSL expression patterns in response to GD3S overexpression in the three cell lines tested. Interestingly, the three cell lines showed different GD2 and GD3 expression patterns after GD3S overexpression, as measured by TLC immunostaining and flow cytometry analysis, i.e., GD2+/GD3+ in MDA-MB231 cells, GD2-/GD3+ in MDA-MB468 cells, and GD2+/GD3- in MCF7 cells. These differential GSL expression patterns provide a reasonable explanation for the variable effects of GD3S overexpression in cancer cells and CSCs. GD3S overexpression also altered expression of several GSL species besides GD2 and GD3. Future studies will address the possible effects of such changes on cancer cell and CSC phenotypes.

Our MS analyses revealed the presence of both a-series (GM1, GM2, and GM3) and b-series (low-level GD1 and GD3) gangliosides in WT MDA-MB231 cells. GD3S transfection led to increased expression of b- and c-series gangliosides. P. Delannoy's group reported that WT MDA-MB231 expressed only a-series gangliosides, mainly GM3 and GM2 [15, 16], and that GD3S-transfected cells accumulated b- and c-series gangliosides. The minor difference between the previous findings and ours may be ascribable to differences in sensitivity of MS analysis and/or GSL extraction methods. We observed that MCF7 expresses both neutral and sialylated GSLs. Following GD3S transfection, these cells also expressed GD2 and the complex ganglioside GD1. Delannoy's group studied specific clones expressing high levels of b- and c-series gangliosides and complex GSLs. They observed accumulation of unusual tetra- and penta-sialylated derivatives of LacCer, GQ3 (II3Neu5Ac4-Gg2Cer), and GP3 (II3Neu5Ac5-Gg2Cer) in GD3S+ clones [35]. In contrast, we observed no GQ3 or GP3 signals in our MS spectra from pooled GD3S-transfectant populations. This apparent discrepancy is likely due to differences between individual clones and pooled populations, or it may be attributed to methods used for cell sampling. Despite these relatively minor differences, both studies show that WT MCF7 express a complex GSL pattern including neutral and sialylated GSLs.

Our combination of approaches to probe association between gangliosides and GFRs (co-IP, IF, immunogold-TEM, and PLA) provides novel and consistent evidence for strong GD3/EGFR association in breast CSCs and breast cancer cell lines. In breast CSCs, the association between GD2 and c-Met was observed but less obvious. Moreover, GD3-positive cell lines (MDA-MB231 and MDA-MB468 with GD3S overexpression) showed strong GD3/EGFR association, while GD2-positive cell lines (MDA-MB231, MCF7 with GD3S overexpression) showed GD2/c-Met association.

The involvement of gangliosides in regulating signal transduction has been reported [36], and many GFRs are either upregulated or downregulated by gangliosides [37]. Signaling kinases and adaptor molecules (p130Cas, paxillin, FAK) have been shown to respond to GD3 upregulation by conferring malignant phenotypes in melanoma and osteosarcoma [14, 38]. Although, a direct connection between these molecules and GD3 has not been demonstrated. J. Wang and R.K. Yu reported that the interaction between GD3 and EGFR maintained EGFR signaling by enhancing cell surface EGFR stability following endocytosis in mouse neural stem cells [12]. Our co-IP and PLA results provide clear evidence for a GD2/c-Met association, and are consistent with observations by P. Delannoy's group that GD2 is involved in c-Met activation and subsequent activation of MEK/Erk and PI3K/Akt signaling pathways, leading to enhanced breast cancer cell migration and proliferation [15, 16].

EGFR mutations have potential application in diagnosis and therapy of various human cancers. Gefitinib is an EGFR kinase inhibitor that disrupts signaling by blocking the EGFR kinase domain, and is therefore effective only in cancers with activating EGFR mutations. EGFR is highly expressed in more than half of TNBCs. However, results of most breast cancer clinical trials using EGFR kinase inhibitors as single agents have been disappointing [39]. Negative results in these cases result mainly from insufficient knowledge regarding the complexity and heterogeneity of breast cancers. Combination therapies that inhibit multiple aberrant pathways have been proposed and applied in many clinical trials designed to optimize anti-tumor efficacy and avoid drug resistance [40].

A subset of TNBC is characterized by constitutive EGFR activation and PTEN loss, which likely mediates resistance to EGFR inhibitors [41]. Among the cell line models we used in this study, MDA-MB468 cells have EGFR amplification and also PTEN deficiency [42]. In our study, sensitivity of MDA-MB468 cells to gefitinib was significantly enhanced when cells were pretreated with lentivirus carrying GD3S shRNA. K. Furukawa's group reported that GD3 was involved in triggering both Erk and AKT signaling pathways in human melanoma cell lines [40]. In another study, combined treatment with gefitinib and the AKT inhibitor PI-103 had a synergistic, anti-proliferative effect on MDA-MB468 cells [39]. GD3S knockdown may therefore interfere with both Erk and AKT signaling pathways simultaneously. This concept would explain the strong suppression effect of combined treatment with gefitinib and GD3S knockdown on growth of PTEN-deficient, EGFR-overexpressing MDA-MB468 cells. Present and previous findings indicate that GD3 is involved in multiple, convergent signaling pathways, and its inhibition provides a basis for effective therapeutic strategies against metastatic breast cancers. Thus, a combination of GD3S knockdown (by shGD3S) and EGFR kinase inhibition (by gefitinib) may be effective against aggressive TNBC breast cancer cells that are resistant to gefitinib alone.

## MATERIALS AND METHODS

### Cell culture

Breast cancer cell lines MCF7, MDA-MB231, and MDA-MB468 were obtained from the American Type Culture Collection (ATCC) and maintained in the recommended culture medium in a 37 °C incubator with 5% CO_2_ atmosphere. HMLE-Twist-ER cells were kindly provided by R. Weinberg (Whitehead Institute; Cambridge, MA, USA) and grown in one part A medium (Mammary Epithelial Growth Medium, Lonza, with10 μg/mL insulin, 10 ng/mL EGF, 0.5 μg/mL hydrocortisone) and one part B medium (Dulbecco's Modified Eagle's Medium, Gibco, with 10% fetal bovine serum (FBS), 100 units/mL penicillin-streptomycin). To induce Twist expression and consequent EMT, these cells were grown in medium with 40 nM 4-hydroxytamoxifen (Sigma) for 12 d. Previous studies by our group and others have clearly shown that Twist-induced EMT of HMLE-Twist-ER cells results in accumulation of cells with CSC properties [17–19]. In the present study, we used this model and considered HMLE-Twist-ER cells cultured in the absence and presence of 4-hydroxytamoxifen as breast non-CSCs and CSCs, respectively.

### GD3S-overexpressing and GD3S-knockdown cell lines

*ST8SIA1* (NM_003034) human cDNA ORF clone (cat. no. RG223851) was from OriGene. A full-length cDNA fragment was PCR-amplified and sub-cloned into a lentiviral vector pLAS2w. Pbsd (National RNAi Core Facility; Taipei, Taiwan) or mammalian expression vector pCMV-Tag2b (Stratagene). A shST8SIA1 clone (small hairpin targeted GD3S; TRCN0000036045; pLKO.1 vector) was from National RNAi Core Facility. Lentivirus production was performed in a HEK293T cell viral package system. Cell lines MDA-MB231 and MDA-MB468 were transduced with GD3S full-length cDNA or GD3S-short hairpin (sh) sequence containing lentivirus with multiplicity of infection (MOI) = 2. MCF7 cells were transfected with recombinant pCMV-Tag 2B vector containing GD3S full-length cDNA using FuGENE 6 transfection reagent. Stable clones were selected with blasticidin (5 μg/mL) for pLAS2w. Pbsd vector, puromycin (1 μg/mL) for pLKO.1 vector, and G418 (500 μg/mL) for pCMV-Tag 2B vector. Antibiotic-resistant clones were pooled to avoid clonal variation.

### Quantitative reverse transcription PCR (qRT-PCR)

Total RNA was extracted using an RNeasy Plus Mini Kit (Qiagen). The first strand of cDNA was prepared from 5 μg RNA using SuperScript III first-strand Synthesis SuperMix (Invitrogen) with random primers, according to the manufacturer's instructions. Real-time qRT-PCR was performed using 200 ng cDNA in a thermal cycler (ABI PRISM 7900 Sequence Detection System; Applied Biosystems) according to the manufacturer's protocol. Relative quantities of mRNAs were determined using the comparative threshold number (ΔΔCt method), with genes for β-actin, GAPDH, and Ups11 as reference genes. Primer sequences for qRT-PCR are listed in Table 1.

**Table 1:** Primer sequences for qRT-PCR

Gene	qRT-PCR primer sequence
GD3S (ST8SIA1)	F: CTGTGGCCGTCAAATAGATG R: AACCTTTGCCGAATTATGCT
E-cadherin	F: TGCCCAGAAAATGAAAAAG R: GTGTATGTGGCAATGCGTT
N-cadherin	F: ACAGTGGCCACCTACAAAG R: CCGAGATGGGGTTGATAAT
Vimentin	F: GAGAACTTTGCCGTTGAAGC R: GCTTCCTGTAGGTGGCAATC T
Fibronectin	F: CAGTGGGAGACCTCGAGAAG R: TCCCTCGGAACATCAGAAAC
Twist	F: GGAGTCCGCAGTCTTACG R: TCTGGAGGACCTGGTAGA
Snail	F: CCTCCCTGTCAGATGAGG R: CCAGGCTGAGGTATTCCT

F, forward primer; R, reverse primer.

### Aldehyde dehydrogenase (ALDH) activity

ALDH activity of various cell lines was quantified using an ALDEFLUOR^®^ assay kit (STEMCELL Technologies), according to the manufacturer's instructions. Cells were harvested, placed in assay buffer, and incubated for 45 min at 37°C to allow intracellular ALDH to convert uncharged ALDH substrate (BAAA) to negatively charged BAA. As a negative control, an aliquot of ALDEFLUOR-stained cells was quenched immediately with 1.5 mM diethylaminobenzaldehyde (DEAB), a specific ALDH inhibitor. Cells were analyzed using the green fluorescence channel (FL1) on a SA3800 spectral cell analyzer (Sony Biotechnology).

### Mammosphere assay

Breast cancer cells (2 **×** 10^3^) were plated in ultralow-attachment 24-well dishes (Costar) in serum-free basal medium supplemented with 1× B-27 and 10 ng/mL basic fibroblast growth factor (Invitrogen). Each well was added with fresh medium without removal of old medium at 2-day intervals. Cells grown under these conditions for 5–7 d at 37°C formed non-adherent spherical clusters (mammospheres), which were counted under a light microscope.

### Wound healing (scratch) assay

Cells grown to a confluent monolayer were gently scratched (wounded) with a pipette tip, washed twice with medium to remove detached cells, and then provided with fresh medium. Cell migration during wound healing was monitored by taking phase contrast images on an inverted microscope with a *Live Cell Imaging* system (model DMi6000, Leica). Wound areas were estimated by analysis of images using the Image J software program. Wound closure percentage was calculated as: [(area of original wound - area of actual wound)/ area of original wound] ×100.

### Cell attachment assay

To assess cell attachment, cells were trypsinized, plated onto flat-bottom tissue culture plates (Falcon) (4 **×** 10^5^ cells/ well), and allowed to adhere for 6 h. Medium was then removed, non-attached cells rinsed away, and attached cells fixed with 4% paraformaldehyde and stained with crystal violet (0.1%).

### Colony formation assay

MDA-MB468 cells were plated in 6-well plates (600 or 300 cells/well), treated with or without gefitinib (Sigma) and/or shGD3S lentivirus (MOI = 10) for 48 h, washed with phosphate-buffered saline (PBS), and medium was replaced by fresh medium. After 10 d incubation, cells were fixed with 70% ethanol, stained with crystal violet, and colony number was counted under a light microscope.

### GSL extraction, HPTLC analysis, and immunostaining

GSLs were extracted as described previously [3]. In brief, 2 **×** 10^8^ cells were extracted by successive sonication in the following four solvents (each 10 mL): (i) chloroform/methanol (CM) (1:1), (ii) isopropanol/hexane/water (IHW) (55:25:20, lower phase), (iii) IHW (55:25:20, lower phase), and (iv) CM (1:1). The combined extracts were evaporated and dissolved in 6 mL CM (2:1). The solution was added with 1 mL water to give CM/water (CMW) 4:2:1, shaken, and allowed to separate into upper and lower phases. The lower phase was added with 3 mL CM/0.1% NaCl (1:10:10), shaken, and allowed to separate into upper and lower phases. This step, known as the Folch partition, was repeated three times. The upper phases were combined, washed with 0.5 mL CM (2:1), evaporated, and solubilized in distilled water, and the resulting solution was applied to a Sep-Pak C18 cartridge (Varian) for desalting.

GSLs were analyzed using HPTLC plates (EMD Bioscience) and developed in a solvent system of CM/0.5% aqueous CaCl_2_ (50:40:10). GSLs were visualized by spraying with 0.5% orcinol in 1 M sulfuric acid. For immunostaining, the plates were dried, fixed with 5% (w/v) poly (isobutyl methacrylate) in hexane/chloroform (9:1), blocked with 3% (w/v) BSA in PBS, probed with anti-GD2 mAb (Clone 14.G2a, #554272, BD Biosciences) or anti-GD3 mAb (R24 clone, ab 11779, Abcam), and incubated with appropriate horseradish peroxidase (HRP)-conjugated secondary Abs. GSLs were detected using an enhanced chemiluminescence (ECL) detection kit (Pierce).

### Mass spectrometric analysis of GSLs

MALDI-TOF MS profiling analyses of permethylated GSLs were performed on a TOF/TOF 5800 system (Sciex; Canada) using 2,5-dihydroxybenzoic acid as matrix (10 mg/mL in 50% acetonitrile). Permethylated derivatives were dissolved in 50% acetonitrile solution. An aliquot of each sample solution was premixed with an equal amount of matrix solution, and spotted on a MALDI plate. Each MALDI-TOF MS spectrum was acquired automatically in 2000 laser shots with random sampling acquisition.

### Flow cytometric analysis

Cells were detached with trypsin, and single cells were washed and resuspended in PBS containing 2% FBS. Cell suspensions were incubated with optimal concentrations of anti-GD2 mAb (Clone 14.G2a, #554272, BD Biosciences) or anti-GD3 mAb (R24 clone, ab 11779, Abcam) for 20 min at 4°C. Cells were washed and then incubated at 4 °C with fluorescence-labeled secondary Ab for 20 min. Control experiments were performed using appropriate isotype controls, or secondary Ab alone. Cells were subjected to flow cytometric analysis using a SA3800 spectral cell analyzer and FlowJo software program.

### Co-immunoprecipitation and immunoblotting

Cells were lysed in 1× RIPA buffer (Cell Signaling Technology) with Halt Protease Inhibitor Cocktail (Thermo Fisher Scientific). The lysate was incubated on ice for 30 min and cleared by centrifugation at 13,000 Rcf for 30 min at 4 °C. Immunoprecipitation was performed with anti-GD2 mAb, anti-GD3 mAb, or normal mouse IgG (sc-2025; Santa Cruz) in the presence of protein A Sepharose (Dynabeads^®^ Protein A, #10002D, Invitrogen) 4 h (or overnight) at 4°C in a rocking incubator. Resulting immune-complexes were subjected to immunoblotting. Blots were probed with specific primary Abs, then incubated with appropriate HRP-conjugated secondary Ab for 1 h. Bands were visualized with ECL reagents (PerkinElmer).

Primary Abs used for immunofluorescence staining were EGFR (ab32562; Abcam), c-Met (sc-10), integrin β1 (sc-9936) (Santa Cruz), anti-fibronectin (rabbit, F1, ab32419, Abcam), anti-vimentin (rabbit, V9, sc-6260, Santa Cruz), anti-N-cadherin (mouse, 32/N-Cadherin, #610921, BD Bioscience), anti-phospho EGFR Tyr1173 (rabbit, 53A5, #4407, Cell signaling), anti-ERK1/2 (rabbit polyclonal, sc-94, Santa Cruz), anti-phospho ERK1/2 Tyr204 (mouse, E4, sc-7383, Santa Cruz), anti-Akt (mouse, 40D4, #2920, Cell signaling), anti-phospho Akt Ser473 (rabbit, D9E, #4060, Cell signaling) and anti-GAPDH (rabbit polyclonal, G9545, Sigma-Aldrich).

### Immunofluorescence cell staining

Cells grown on glass coverslips were fixed in 4% paraformaldehyde in PBS for 15 min at room temperature (RT), and permeabilized with 0.5% Triton X-100 in PBS for 5 min. Fixed cells were blocked with 5% BSA in PBS for 30 min, incubated overnight at 4°C with primary Ab, washed, incubated 1 h at RT with secondary Ab, and counterstained with DAPI (Pharmingen). Coverslips were mounted with glycerol mounting medium (Dako) and sealed with clear nail polish. Fluorescence images were obtained by confocal immunofluorescence microscopy (model TCS SP8; Leica).

Primary Abs used for immunofluorescence staining were anti-GD2 (mouse, Clone 14.G2a, #554272, BD Biosciences), anti-GD3 (mouse, clone R24, ab 11779, Abcam), anti-EGFR (rabbit, ab32562; Abcam), and anti-c-Met (rabbit, sc-10, Santa Cruz). Secondary Abs used were Alexa555-conjugated donkey anti-mouse IgG and donkey anti-rabbit IgG (Jackson ImmunoResearch) and Alexa488-conjugated donkey anti-rabbit IgG and donkey anti-mouse IgG (Invitrogen).

### Immunogold labeling and transmission electron microscopy (TEM)

Cells were grown on glass coverslips, placed upside-down against Formvar/carbon-coated nickel grids coated with poly-L-lysine, adhered to the grids by light pressure (2 s) on the top of the coverslip, washed with KOAc buffer, and fixed with 4% paraformaldehyde and 0.1% glutaraldehyde (Electron Microscopy Sciences). Free aldehyde groups were quenched by 20 mM glycine. Grids were blocked with 5% donkey serum (Jackson ImmunoResearch Labs), incubated overnight at 4°C with primary Abs (mouse anti-GD2, mouse anti-GD3, rabbit anti-EGFR, and rabbit anti-c-Met, as described above), washed with 0.1% BSA/PBS, and incubated 1 h, at RT with secondary Abs (colloidal gold conjugated polyclonal Abs (6 nm colloidal gold AffiniPure goat anti-rabbit IgG (H+L)) or 12 nm colloidal gold AffiniPure goat anti-mouse IgG (H+L), Jackson ImmunoResearch). Immunogold labeled grids were fixed with 1% glutaraldehyde, washed with double-distilled water, stained with 3% uranyl acetate for 30 min, washed with water, and examined by TEM (model H-7500; Hitachi) at 75 kV.

### *In situ* proximity ligation assay (PLA)

Interactions among gangliosides and GFRs were assessed using an *in situ* PLA kit (Duolink) according to the manufacturer's instructions. In brief, cells (2 × 10^4^) grown on 8-well slides (ibidi) were fixed in 4% paraformaldehyde in PBS for 15 min at RT, blocked with Duolink blocking solution at 37°C for 30 min, washed with PBS, incubated overnight at 4°C with primary Abs (mouse anti-GD2, mouse anti-GD3, rabbit anti-EGFR, and rabbit anti-c-Met, as described above), washed with PBS, incubated 1 h at 37°C with secondary Ab (anti-mouse PLA-plus probe or anti-rabbit PLA-minus probe; Duolink; dilution 1:50), washed twice (5 min each time) with Duolink Wash buffer A, added with Duolink ligation mixture, incubated 30 min at 37°C, washed twice with Wash buffer A, added with Duolink amplification mixture and Polymerase, subjected to amplification reaction for 100 min at 37°C, washed twice with Wash buffer B and once with 0.1× Wash buffer B, and mounted with Duolink *In Situ* Mounting Medium with DAPI. Fluorescence dot images were obtained by confocal immunofluorescence microscopy as above.

### Determination of IC50 (half maximal inhibitory concentration) value

IC50 values of gefitinib in breast cancer cell lines were assessed by alamarBlue^®^ Assay (Thermo Fisher). Cells were harvested with trypsin, plated in 96-well plates (1.5 × 10^3^ cells/ well), left for 24 h to recover, and treated with gefitinib at various concentrations (1.25 to 20 μM) for 48 h. AlamarBlue dye was added to each well (10% of well volume) and incubated 4 h. Fluorescence was measured on a SpectraMax M2 microplate reader (Molecular Devices; USA) at excitation wavelength 560 nm, emission wavelength 590 nm. Growth inhibition rate was calculated as (1 - mean fluorescence value of drug-treated samples/mean fluorescence value of untreated controls) × 100%.

### Mouse xenograft model and *in vivo* tumorigenicity

MDA-MB468 cells (5 × 10^6^) stably expressing control lentiviral construct (shLuc) or GD3S shRNA (shGD3S) were harvested and mixed with Matrigel for subcutaneous injection into the mammary fat pads of 5 week old female NOD/SCID/IL2Rgamma null (NSG) mice. Once the tumors were palpable, the mice in each group were randomly allocated to treatment groups with four animals each: (1) no treatment (control) or (2) gefitinib (twice weekly by oral gavage at dose of 150 mg/kg). Tumor growth was monitored by caliper measurements of two perpendicular diameters three times weekly, and the volume of the tumor was calculated with the formula V = (length×width^2^) × (π/6). NSG mice were purchased from The Jackson Laboratory, and bred in our animal facility under Specific-pathogen-free conditions. All procedures were performed in compliance with the Regulations for the Institutional Animal Care and User Committee of Chang-Gung University.

### Statistical analysis

All experiments were performed in triplicate and repeated three times, and data are reported as mean ± SEM. Statistical analysis was performed using the Prism^®^ 5 software program (GraphPad). Data were analyzed by one-way ANOVA followed by Newman-Keuls multiple comparison *post hoc* test to compare all groups with the control group, or by unpaired Student's *t*-test to compare designated pairs of groups. IC50 data was analyzed by one-way ANOVA followed by Fisher's least significant difference method. Differences were considered significant at *p* < 0.05.

## Abbreviations

GSL: glycosphingolipid
GD3S: GD3 synthase
EMT: epithelial-mesenchymal transition
CSC: cancer stem cell
TNBC: triple-negative breast cancer
ER: estrogen receptors
PR: progesterone receptors
HER2: human epidermal growth factor receptor 2
HPTLC: high performance thin layer chromatography
MALDI-TOF MS: matrix-assisted laser desorption/ionization time-of-flight mass spectrometry
mAb: monoclonal antibody
EGFR: epidermal growth factor receptor
PLA: proximity ligation assay
GFR: growth factor receptor
